# Bilateral Chilblain-like Lesions of the Toes Characterized by Microvascular Remodeling in Adolescents During the COVID-19 Pandemic

**DOI:** 10.1001/jamanetworkopen.2021.11369

**Published:** 2021-06-10

**Authors:** Valentina Discepolo, Andrea Catzola, Luca Pierri, Massimo Mascolo, Francesca Della Casa, Maria Vastarella, Grace Smith, Antonio Travaglino, Alessandra Punziano, Paola Nappa, Stefania Staibano, Eugenia Bruzzese, Gabriella Fabbrocini, Alfredo Guarino, Maria Alessio

**Affiliations:** 1 Department of Translational Medical Sciences, Section of Pediatrics, University of Naples Federico II, Naples, Italy; 2 Department of Advanced Biomedical Sciences, Pathology Unit, University of Naples Federico II, Naples, Italy; 3 Department of Translational Medical Sciences, Section of Clinical Immunology, University of Naples Federico II, Naples, Italy; 4 Department of Clinical Medicine and Surgery, Section of Dermatology, University of Naples Federico II, Naples, Italy; 5 Laboratory of Pathology, Center for Cancer Research, National Cancer Institute, National Institutes of Health, Bethesda, Maryland

## Abstract

**Importance:**

Chilblain-like lesions have been one of the most frequently described cutaneous manifestations during the COVID-19 pandemic. Their etiopathogenesis, including the role of SARS-CoV-2, remains elusive.

**Objective:**

To examine the association of chilblain-like lesions with SARS-CoV-2 infection.

**Design, Setting, and Participants:**

This prospective case series enrolled 17 adolescents who presented with chilblain-like lesions from April 1 to June 30, 2020, at a tertiary referral academic hospital in Italy.

**Main Outcomes and Measures:**

Macroscopic (clinical and dermoscopic) and microscopic (histopathologic) analysis contributed to a thorough understanding of the lesions. Nasopharyngeal swab, serologic testing, and in situ hybridization of the skin biopsy specimens were performed to test for SARS-CoV-2 infection. Laboratory tests explored signs of systemic inflammation or thrombophilia. Structural changes in peripheral microcirculation were investigated by capillaroscopy.

**Results:**

Of the 17 adolescents (9 [52.9%] male; median [interquartile range] age, 13.2 [12.5-14.3] years) enrolled during the first wave of the COVID-19 pandemic, 16 (94.1%) had bilaterally localized distal erythematous or cyanotic lesions. A triad of red dots (16 [100%]), white rosettes (11 [68.8%]), and white streaks (10 [62.5%]) characterized the dermoscopic picture. Histologic analysis revealed a remodeling of the dermal blood vessels with a lobular arrangement, wall thickening, and a mild perivascular lymphocytic infiltrate. SARS-CoV-2 infection was excluded by molecular and serologic testing. In situ hybridization did not highlight the viral genome in the lesions.

**Conclusions and Relevance:**

This study delineated the clinical, histologic, and laboratory features of chilblain-like lesions that emerged during the COVID-19 pandemic, and its findings do not support their association with SARS-CoV-2 infection. The lesions occurred in otherwise healthy adolescents, had a long but benign course to self-resolution, and were characterized by a microvascular remodeling with perivascular lymphocytic infiltrate but no other signs of vasculitis. These results suggest that chilblain-like lesions do not imply a concomitant SARS-CoV-2 infection. Ongoing studies will help clarify the etiopathogenic mechanisms.

## Key Points

**Question:**

Are chilblain-like lesions of the toes associated with SARS-CoV-2 infection or is the association merely temporal?

**Findings:**

This case series of 17 adolescents found that chilblain-like lesions of the toes emerged during the COVID-19 pandemic in otherwise healthy adolescents without signs of SARS-CoV-2 infection or other inflammatory, autoimmune, or thrombophilic phenomena.

**Meaning:**

These results suggest that chilblain-like lesions are not associated with systemic or localized SARS-CoV-2 infection.

## Teaser

This case series examines the association of chilblain-like lesions with SARS-CoV-2 infection.

## Introduction

SARS-CoV-2 was first identified in January 2020. Since then, COVID-19 disease has widely spread worldwide, becoming a pandemic emergency. Unlike adults, children are more mildly affected by SARS-CoV-2 for reasons that have not been fully elucidated.^1^ The most frequent symptoms in the pediatric population include fever (80%), cough (50%), rhinorrhea (27%), dyspnea (9%), sore throat (5%), and fatigue (2%),^2^ but diarrhea,^2,3^ dysgeusia, and anosmia (8%-13%) also occur. Numerous cutaneous manifestations have been reported during the pandemic, including extremely protean pictures, such as erythematous, varicelliform or morbilliform rash, and urticaria.^4,5,6,7,8,9,10^ However, a causative role of SARS-CoV-2 has not been confirmed, and the pathophysiologic mechanisms remain unknown.^6^

Cases of acral lesions resembling classic chilblains have been reported worldwide at a higher rate than before the pandemic,^10,11^ becoming one of the most commonly reported cutaneous manifestations during the pandemic. Also known as chilblain-like lesions or COVID toes, these acral lesions are described as erythematous to purple purpuric macules, papules, and/or vesicles that predominantly involve the feet and, to a lesser extent, the hands.^12,13^ Unlike other cutaneous findings, chilblain-like lesions tend to mostly affect patients without systemic or evident COVID-19 symptoms^14^; in fact, patients with such eruptions are less likely to have severe disease.^15^ SARS-CoV-2 infection remains unconfirmed in many patients with these lesions. Different pathogenic hypotheses have been advanced, including a delayed cytokine-mediated inflammatory response induced by SARS-CoV-2,^16^ viral-induced endothelial changes, obliterative microangiopathy, and coagulation abnormalities,^17^ although coagulation abnormalities have been related to acroischemia rather than COVID toes. However, none of these hypotheses have been convincingly confirmed, and a causal association with the virus has not been demonstrated. On the other hand, exposure to cold temperatures, typical of classic perniosis, has not been consistently reported, probably because of different geographic locations. This study examines the association of chilblain-like lesions with SARS-CoV-2 infection among pediatric patients

## Methods

### Study Design and Patients

In this cases series, we prospectively enrolled 17 pediatric patients referred by family pediatricians for chilblain-like lesions to our Pediatric Infectious Disease and Rheumatology Unit at the University of Naples Federico II, Naples, Italy, designated as the Regional COVID-19 Pediatric Hub. Exclusion criteria included other nonacral skin lesions. Seventeen children were enrolled from April 1 to June 30, 2020 (Table). At enrollment, all patients underwent a thorough clinical examination, laboratory workup, dermatologic evaluation (including dermoscopy),^18^ and a skin biopsy to investigate the histologic features and the presence of SARS-CoV-2. All patients underwent capillaroscopy to exclude systemic microvascular alterations.^19,20^ A follow-up appointment was set at 4 weeks after enrollment and included clinical, laboratory, and dermatologic evaluation. All patients underwent a nasopharyngeal swab for SARS-CoV-2 molecular testing at enrollement and quantitative IgM and IgG serologic testing against SARS-CoV-2 at enrollement and follow-up to investigate the association with SARS-CoV-2 infection. The study protocol was approved by the University of Naples Ethical Committee, and all enrolled patients provided written informed consent. All data were deidentified. The study followed the reporting guideline for case series.

**Table.** Demographic and Clinical Data of the Study Patients

Patient No.	Patient sex	Patient age, y	Localization	Bilateral	Past chilblains	Prechilblains symptoms	COVID-19–related symptoms	SARS-CoV-2 nasopharyngeal swab result	Family history of SARS-CoV-2	Relevant personal medical history	Family history of autoimmunity
1	F	12.5	Foot	Yes	No	URTI	None	Negative	Yes	None	No
2	M	13.0	Foot	No	No	URTI	None	Negative	No	None	Thromboangiitis obliterans
3	M	11.0	Foot	Yes	No	None	None	Negative	No	None	Hemolytic anemia during pregnancy
4	F	12.5	Foot	Yes	No	None	None	Negative	No	None	Hemolytic anemia during pregnancy
5	F	12.8	Foot	Yes	No	URTI	None	Negative	No	None	Autoimmune thyroiditis
6	F	14.5	Hand and foot	Yes	No	None	None	Negative	Not confirmed	None	No
7	F	12.8	Foot	Yes	No	None	None	Negative	Not confirmed	None	No
8	M	14.5	Foot	Yes	No	None	None	Negative	No	None	No
9	F	10.5	Hand and foot	Yes	No	None	None	Negative	No	None	No
10	M	13.4	Foot	Yes	No	None	None	Negative	No	Henoch-Schönlein purpura	No
11	M	14.0	Foot	Yes	No	Rhinitis	None	Negative	No	None	Autoimmune thyroiditis
12	M	14.5	Foot	Yes	No	None	None	Negative	No	None	Autoimmune thyroiditis and IBD
13	M	14.3	Foot	Yes	No	None	None	Negative	No	None	Autoimmune thyroiditis
14	M	13.2	Foot	Yes	No	None	None	Negative	No	None	No
15	M	13.5	Foot	Yes	No	None	None	Negative	No	None	No
16	F	15.1	Foot	Yes	No	None	None	Negative	No	None	No
17	F	9.0	Foot	Yes	No	URTI and GI symptoms	None	Negative	Yes	None	Autoimmune thyroiditis

Abbreviations: GI, gastrointestinal; IBD, inflammatory bowel disease; URTI, upper respiratory tract infection.

### Laboratory Workup

Laboratory tests were performed at both enrollement and follow-up. They included complete blood cell count; complete metabolic panel; coagulation; D-dimer; circulating immune complexes (circulating immune complex 1Q and circulating immune complex 3d); complement factors (C3 and C4); inflammatory markers, including procalcitonin, C-reactive protein, erythrocyte sedimentation rate, and ferritin; autoimmune panel, including antinuclear antibodies and extractable nuclear antigen profile; and total immunoglobulins (total IgA, IgM, and IgG).

### SARS-CoV-2 Laboratory Tests

Real-time reverse transcriptase–polymerase chain reaction of nasopharyngeal swabs was performed at the centralized laboratory of the University of Naples Federico II. Quantitative SARS-CoV-2 serologic testing was performed by chemiluminescent immunoassay, which reported a sensitivity of 79% and a specificity of 97.5% for IgM (Snibe Diagnostics) and a sensitivity of 100% and a specificity of 99.6% for IgG (Abbott Laboratories).

### Macroscopic (Clinical and Dermoscopic) and Microscopic (Histopathologic) Analysis

Patients were examined using video dermoscopy (DermaView) by 3 independent physicians (M.V., P.N., and G.F.) according to the International Dermoscopy Society’s expert consensus.^18^ In addition, histopathologic analysis was performed with a 3-mm skin punch biopsy taken at enrollement from a representative area of each patient. Then 4-mm-thick formalin-fixed, paraffin-embedded sections were stained with hematoxylin and eosin. Immunohistochemistry was performed as previously described^21^ with anti-CD3 and anti-CD31 antibodies to highlight the lymphocytic infiltrate and the endothelium, respectively.

In situ hybridization (RNAscope 2.5 HD Assay-BROWN; Advanced Cell Diagnostics) was used according to the manufacturer’s instructions to perform in situ hybridization on 5-μm-thick formalin-fixed, paraffin-embedded skin sections. *POLR2A* (OMIM 180660) messenger RNA labeling was used as a positive control. Slides were deparaffinized and air dried followed by hydrogen peroxide drying. Slides were submerged in fresh 1× target retrieval (Advanced Cell Diagnostics) at boiling temperature for 15 minutes, washed in deionized water followed by 100% ethanol, and air dried. Protease (Protease Plus; Advanced Cell Diagnostics) was applied for 20 minutes in a hybridization oven at 40 °C. Probes were incubated for 2 hours in a hybridization oven. Signal amplification reagents AMP1 to AMP6 were applied sequentially and incubated in a hybridization oven. 3,3′-Diaminobenzidine detection reagent was incubated for 10 minutes in a hybridization oven. Samples were counterstained with hematoxylin, rinsed with tap water, placed in 0.02% ammonia-water, rinsed with tap water, dehydrated in graded alcohols, treated with xylene, and coverslipped. Videocapillaroscopy^19,20^ of the fingers was also performed using a high-magnification lens (original magnification ×200) to allow for better visualization of the morphologic features of the capillaries.

### Statistical Analysis

For descriptive statistics, data are presented as mean (SD) for continuous values with gaussian distribution, as median and range for continuous values with non-Gaussian distribution, and as counts and percentages for categorical variables. Analyses were performed using GraphPad Prism, version 7.0 (GraphPad Software Inc).

## Results

### Clinical Features

**Figure 1.**
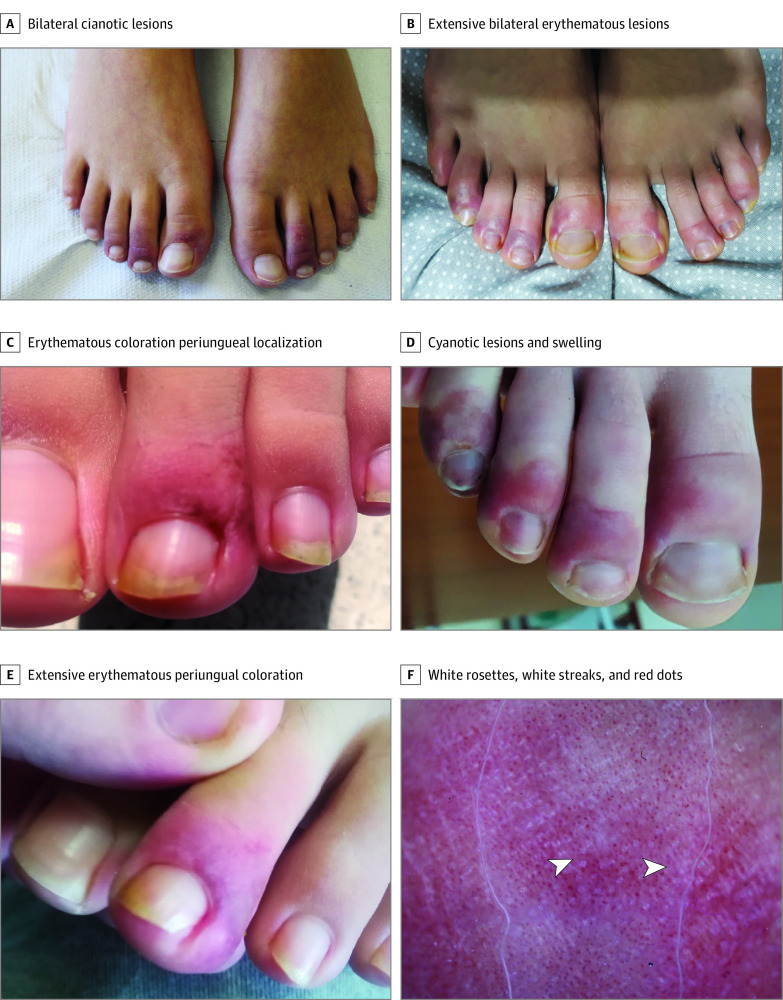
Bilateral Periungual Erythematous Lesions of the Toes in Adolescents During the COVID-19 Pandemic Representative pictures of the skin lesions highlighting the bilateral distribution (A-B), the periungual localization (A-E), and erythematous coloration (C-E). F, Dermoscopic image relative to the lesions pictured in panel D characterized by a peculiar triad, including white rosettes (left arrowhead), white streaks (right arrowhead), and red dots over an erythematous background.

All 17 enrolled patients were healthy-appearing adolescents (9 [52.9%] male; median [interquartile range] age, 13.2 [12.5-14.3] years) who presented with erythematous or cyanotic acral lesions that involved the periungual area of the toes (Figure 1A-E). Lesions were bilaterally distributed (Figure 1A and B) in 16 patients (94.1%), and the skin area of the heels was involved in 7 patients (41.2%) (eFigure 1A-C in the Supplement). Ulceration complicated 1 case during the active phase of the disease (eFigure 2 in the Supplement), whereas desquamation developed over time in 3 cases (17.6%) (eFigure 2E in the Supplement). Concurrent involvement of the fingers was observed in only 2 patients (11.8%) (Table).

Self-administered therapies included topical antibiotics and/or corticosteroids in 4 of 17 patients (23.5%), disinfectants in 4 of 17 patients (23.5%), and antifungal agents in 3 of 17 patients (17.6%), whereas systemic antibiotics or corticosteroids were used rarely (2 of 17 patients [11.8%]). None of the therapies substantially changed the course of the lesions, which was characterized initially by a gradual fading of the erythema and lastly by a gradual fading of the cyanosis, whereas the localization and distribution of the lesions remained unchanged over time (Figure 1; eFigure 2 in the Supplement). At onset, the lesions consisted of erythematous-purpuric papules and macules, whereas in the later stages they appeared as blurred, rosaceous, erythematous maculae with postinflammatory hyperpigmentation. In contrast to Raynaud phenomenon and perniosis, no discoloration of the extremities preceded the erythema and cyanosis, suggesting that vasospasm did not play a major role. The duration of the lesions was extremely variable, ranging from 49 to 145 days (eTable 1 in the Supplement); however, at follow-up, all patients had full resolution of the lesions.

The onset of lesions was not preceded by any prodromes. The most commonly associated signs and symptoms were pain, swelling (eFigure 1D in the Supplement), and pruritus (9 patients [52.9%]), whereas a burning sensation of the involved areas was reported by 4 patients (23.5%). Concomitant sore throat, cough, diarrhea, and dysgeusia were only sporadically reported. Subjective symptoms disappeared along with the clinical resolution.

A medical history of idiopathic perniosis and/or Raynaud phenomenon was not reported by any of the adolescents. Patients were interviewed in the presence of their parents, and either the patient or the parents could answer. Most often the parents answered. Similarly, a personal history of chronic autoimmune diseases was not reported by any of the patients. A positive family history of autoimmunity was noted in 6 patients (35.3%) (Table).

### Laboratory Tests

All patients had normal laboratory test results, except for mild elevation of the complement C3 fraction (eTable 2 in the Supplement) found in all patients at enrollment (mean [SD], 123 [19] mg/dL; upper limit of the reference range, 95 mg/dL [to convert to grams per liter, multiply by 0.01]) and in 10 of the 17 tested patients at follow-up (mean [SD], 116 [17] mg/dL). The increase in C3 was mild and persisted at follow-up, despite the clinical resolution of the lesions, thus indicating a minor role in their onset. Of importance, the results of the laboratory tests performed (eTable 2 in the Supplement) allowed for exclusion of an ongoing systemic inflammatory process and ongoing autoimmune phenomena. Moreover, normal levels of D-dimer, fibrinogen, and platelets excluded a procoagulant state.

### Dermoscopy

All but 1 of the enrolled patients (94.1%) underwent dermoscopic evaluation at enrollment, whereas only 4 (23.5%) received a second assessment. In line with previous reports,^22^ a dermoscopic triad almost invariably characterized the lesions, including red dots (16 of 16 patients [100%]), white rosettes (11 of 16 patients [68.8%]), and white streaks (10 of 16 patients [62.5%]) on an erythematous background (Figure 1F; eTable 1 in the Supplement). Red dots often appeared as dotted and comma-shaped congested vessels (9 of 16 patients [56.2%]), surrounding the rosettes in the early stage of the lesions. In later stages, red dots were still observed, whereas the rosettes disappeared. Although inconstantly found in inflammatory cutaneous conditions, these 3 signs do not characterize the dermoscopic picture of perniosis, suggesting a distinct disease process.

### Histologic Analysis

**Figure 2.**
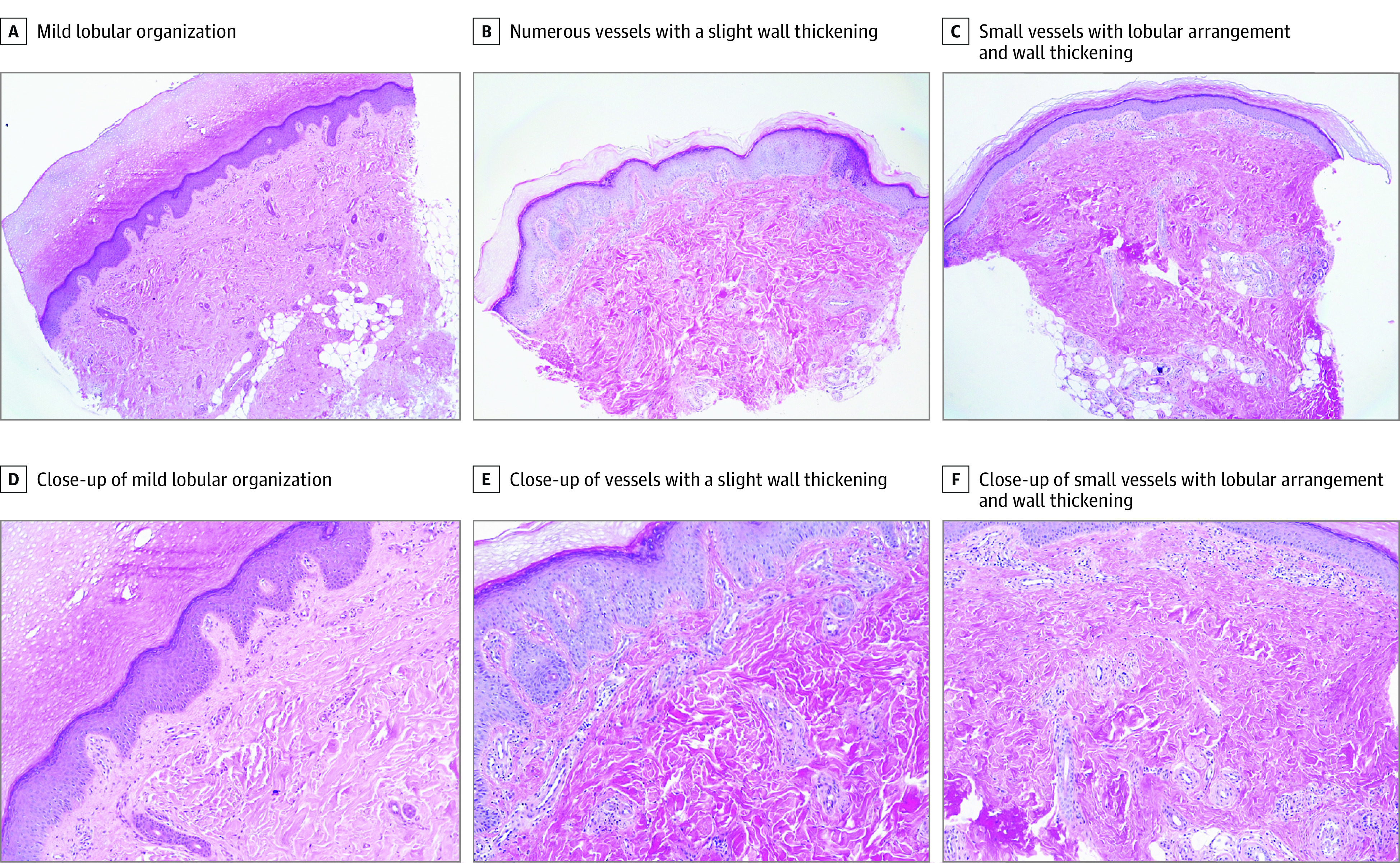
Microvascular Remodeling Characterized by Lobular Organization of the Vessels and Wall Thickening Hematoxylin-eosin immunohistochemistry images from 3 representative patients (each column includes images from the same case). Original magnification ×4 (A-C); original magnification ×10 (D-F).

**Figure 3.**
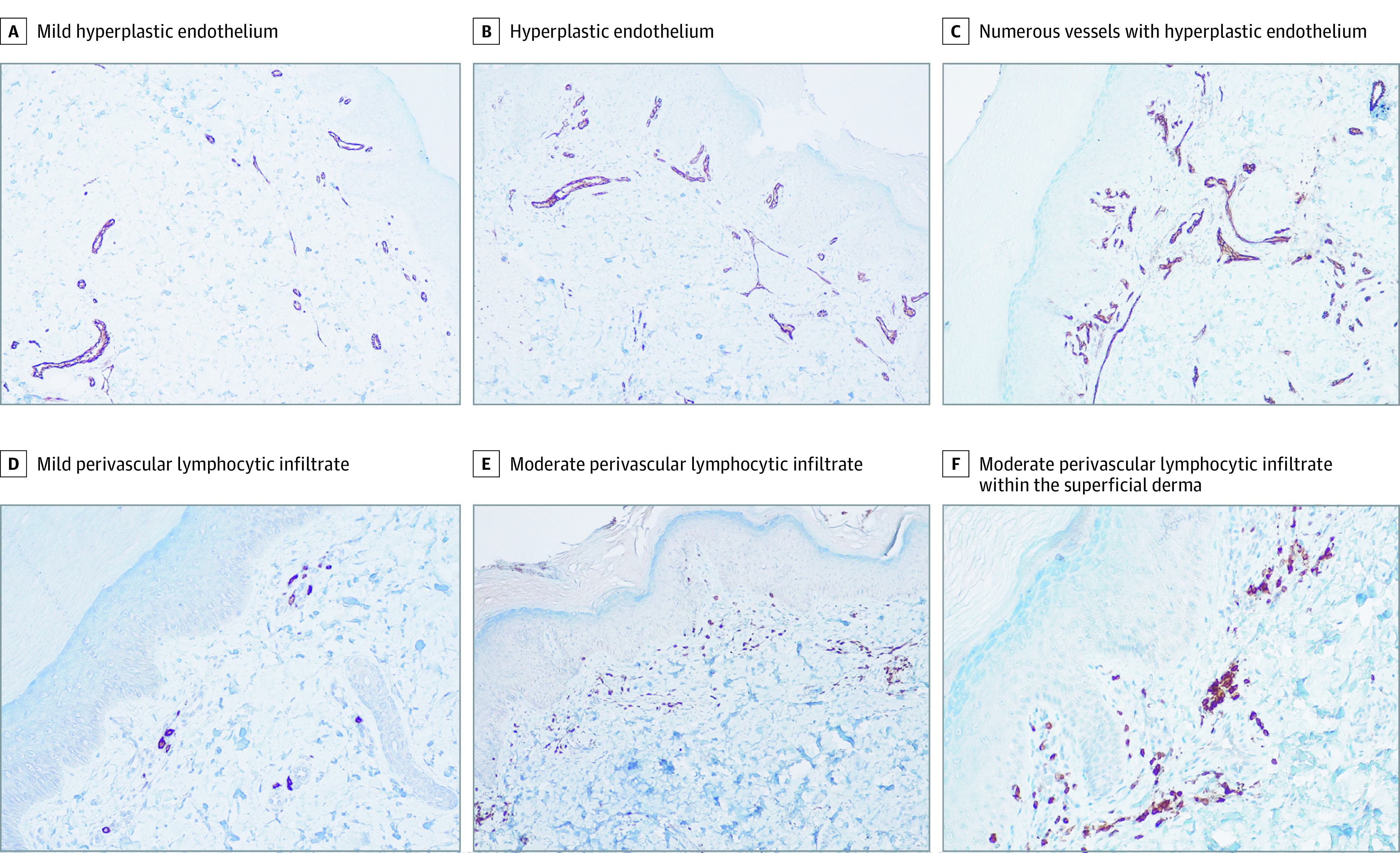
Microvascular Remodeling Featuring Endothelial Hyperplasia and a Mild Perivascular Lymphocytic Infiltrate Immunohistochemistry images from 3 representative patients (original magnification ×10). As in Figure 2, each column includes images from the same case. A-C, Endothelial cells are highlighted by anti-CD31 staining. D-F, Lymphocytes are highlighted by anti-CD3 staining.

A punch biopsy of the involved skin was taken in 12 of 17 cases (70.6%). Histologic analysis showed a slight remodeling and increased number of the dermal blood vessels with a tendency to a lobular arrangement (Figure 2; eTable 3 in the Supplement). Immunohistochemical analysis revealed a slight wall thickening with endothelial hyperplasia (anti-CD31 staining) (Figure 3A-C) associated with mild lymphocytic perivascular infiltrate (anti-CD3 staining) (Figure 3D-F). Interestingly, the histologic features evolved from a low-grade lymphocytic infiltration (earlier lesions) to a reorganization of the vascular structures, which appeared lobular and hyperplastic (advanced lesions). In contrast with idiopathic perniosis (eFigure 3A in the Supplement), none of the patients had edema of the papillary derma or fluffy edema of the vessels. Moreover, despite several biopsy specimens having been taken at the onset of the lesions, none of them revealed the eosinophilic or neutrophilic infiltrate typical of early stages of perniosis. In line with a milder histologic picture than classic perniosis, no vacuolar changes of the basal layer or signs of leukocytoclastic vasculitis (eFigure 3B in the Supplement) were found.

### Association With SARS-CoV-2 Infection

Despite most chilblain-like lesions having been described in asymptomatic or minimally symptomatic SARS-CoV-2 cases,^14^ because all of the patients were enrolled during the first wave of the COVID-19 pandemic, a thorough interview exploring the occurrence of SARS-CoV-2–suggestive symptoms was conducted. Those symptoms were only episodically reported, with sore throat reported in 2 of the 17 patients (11.8%) and cough, dysgeusia, and diarrhea reported in 1 of the 17 patients (5.9%). Three weeks before the onset of the skin lesions, upper respiratory tract symptoms were reported by 3 patients (17.6%) and diarrhea by 2 patients (11.8%). At enrollment, 2 patients reported previous contact with a person with a confirmed positive SARS-CoV-2 case, whereas 2 patients had contact with a suspected but not a confirmed case (Table). Nevertheless, molecular test results were negative for SARS-CoV-2 in all patients. Similarly, quantitative determination of SARS-CoV-2–specific IgM and IgG antibodies was negative in all patients at enrollment and follow-up.

**Figure 4.**
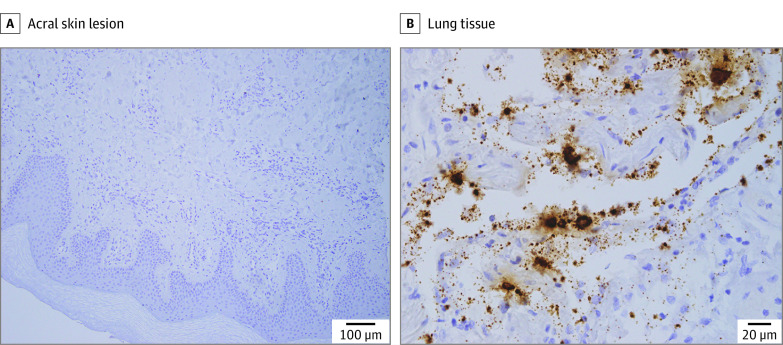
Absence of SARS-CoV-2 Viral Genome in the Skin Lesions In situ hybridization was performed on skin biopsies of the acral lesions (1 representative image, scale bar = 100 μm) (A) and lung tissue as a positive control (B) from a SARS-CoV-2–positive case (scale bar = 20 μm).

Because some authors^23^ ascribed chilblain-like lesions to a local SARS-CoV-2 infection, to further explore its potential role, we investigated the presence of SARS-CoV-2 RNA in the skin lesions by in situ hybridization, and in line with the laboratory tests, we did not find the viral genome in the lesions of any of the enrolled adolescents (Figure 4). Altogether these data do not support the association of the lesions with SARS-CoV-2 infection.

### Capillaroscopy

To exclude the presence of signs suggestive of a systemic inflammatory process of the acral microvascular districts, we performed capillaroscopy at enrollment in 15 of the 17 patients (88.2%) (eTable 4 and eFigure 4 in the Supplement). In 80% of the patients, capillaroscopic results were either completely normal (6 of 15 [40.0%]) or showed rare ectasias (6 of 15 [40.0%]), supporting the lack of a systemic inflammatory process. A winding organization of vessels (3 of 15 patients [20.0%]) or more frequent ectasias (3 of 15 patients [20.0%]) were rarely described.

## Discussion

This case series of otherwise healthy adolescents delineated a clinical picture that emerged during the COVID-19 pandemic of chilblain-like lesions of the toes associated with microvascular remodeling with a long but benign course to self-resolution not associated with signs of SARS-CoV-2 infection. These skin lesions were characterized by erythematous or cyanotic acral lesions typically bilaterally localized at the distal extremities, with invariable involvement of the periungual area of the toes. The onset was often associated with pain, swelling, and pruritus. A dermoscopic triad, including red dots, white rosettes, and white streaks, was invariably reported, in line with previous findings.^22^

Classic erythema pernio is an inflammatory vascular response of the superficial dermis of the proximal extremities, typically occurring in middle-aged women after exposure to cold or after a sudden temperature change.^8^ Together with their occurrence in childhood and distal localization, another unique feature of the lesions observed in this case series was the involvement of the heels, which is absent in classic chilblains. Whether walking barefoot at home, which has occurred more frequently during the COVID-19 lockdown, contributed to the development of lesions remains a hypothesis.^24^

Histopathologic analysis revealed signs of dermal vascular remodeling, including a tendency toward lobular arrangement, slight wall thickening, and variable endothelial hyperplasia. In contrast to idiopathic perniosis,^25^ none of the patients had dermal edema or eosinophilic infiltrate, as found in the early stages of perniosis and in previously reported cases of COVID toes.^26^ In line with this adult case series, the perivascular lymphocytic infiltrate and endothelial cell swelling were 2 of the most commonly reported features, although parakeratosis or vacuolar changes of the basal layer, which are commonly described in autoimmune chilblains, were not observed. The milder histologic findings in this case series might have been associated with the younger age of the patients and suggest a chronic rather than an acute inflammatory process, resulting in tissue remodeling that resembles vascular stasis more than vasculitis. Accordingly, capillaroscopy did not reveal signs of impaired microcirculation in the upper limbs, in contrast to systemic vasculitis.

Chilblains might also occur secondary to underlying conditions, such as connective tissue diseases, cryopathies, neoplastic diseases, blood hyperviscosity, genetic diseases (ie, familial chilblains lupus or Aicardi-Goutières syndrome), anorexia, and malnutrition.^25^ However, the patients in this case series did not report a personal history of autoimmunity and did not have any laboratory signs of underlying autoimmune, inflammatory, or proliferative conditions.

Of interest, vascular deposits of C3, together with IgA and IgM, were previously reported in 5 patients who presented with chilblain-like lesions.^26^ In contrast to a depletion of circulating complement, as expected in association with vascular deposits, a mild elevation of the C3 fraction was seen in all patients and persisted during follow-up. Despite this finding seeming nonspecific, the role of the complement in the genesis of the lesions remains to be elucidated. Hypercoagulability, which might be associated with acroischemia more than chilblain-like lesions, was also excluded.

The temporal association between the COVID-19 pandemic and the increasing number of chilblain-like lesions, to the point that the media now refers to it as COVID toes, suggests that this condition could be a cutaneous sign of SARS-CoV-2 disease.^25,27,28^ However, data on the association with SARS-CoV-2 are controversial.^29,30^ The first case series failed to perform SARS-CoV-2 testing in all patients.^8,12,31^ In line with several studies,^32,33^ in this case series, molecular, serologic, and tissue data did not show signs of current or past SARS-CoV-2 infection in any of the patients. A French study^27^ reported similar skin lesions in adults enrolled during April 2020. In contrast to the findings of this case series, the French investigators observed a more frequent bullous and necrotic evolution and an increase in antinuclear antibodies in approximately 20% of patients and in D-dimer levels in 60% of patients. In the previous study,^27^ histopathologic analysis revealed a more intense lymphocytic infiltration, probably because only the most severe cases underwent skin biopsy. Whether these differences might be attributable to patient age or to a distinct pathogenic mechanism remains elusive. The authors did not identify any patients with SARS-CoV-2, yet they hypothesized, in line with other authors,^30^ that the lesions might have been the result of a delayed immune response to SARS-CoV-2 exposure. In contrast with this hypothesis, all adolescents included in the present case series also had negative IgM- and IgG-specific serologic test results at 4 weeks of follow-up. Moreover, to rule out a direct role of the virus in the development of the lesions, the current study performed in situ hybridization of the skin biopsy specimens and systematically failed to detect the viral genome in the skin lesions of the patients, further demolishing the causative role of SARS-CoV-2. This finding is in contrast to the hypothesis of a local endothelial or cutaneous infection proposed by some authors^23,26,34^ but in line with previous studies^22,35^ that failed to detect SARS-CoV-2 in chilblain-like lesions by in situ hybridization or immunohistochemical analysis.

Despite the increasing incidence of COVID-19 cases in Campania in Southern Italy, from 10 to 20 per 100 000 population during the first wave of the pandemic to 75 per 100 000 population during the second wave,^36^ only 3 new chilblain-like cases were reported after June 2020. That only 3 new cases were reported during the highest peaks of the pandemic further supports a lack of association with SARS-CoV-2 infection; in addition, none of these patients tested positive for SARS-CoV-2. The fact that all 3 cases during the second wave occurred in winter months (November 2020, January 2021, and March 2021) suggests that exposure to the cold might, at least in some cases, trigger the skin lesions. In line with this hypothesis, 7 of the enrolled adolescents in this case series (41.2%) relapsed during the winter months while again testing negative for SARS-CoV-2.

### Limitations

This study has limitations, the main limitation being the small sample size. In addition, the study design did not allow risks to be calculated or cause-effect relationships to be established.

## Conclusions

This case series included 17 adolescents who presented with chilblain-like lesions of the toes associated with microvascular remodeling but no evidence of current, past, or local SARS-CoV-2 infection. Lifestyle changes, including reduced physical activity and avoidance of footwear at home (related to the lockdown restrictive measures imposed by the government), might have contributed to the onset of the lesions. The increased emphasis by the media on the pandemic and the associated emerging clinical manifestations might have contributed to the increase in attention on milder cases that would have not undergone clinical examination in the prepandemic era. Whether genetic predisposing factors contribute to the onset of these lesions in selected adolescents remains to be explored. Larger ongoing studies will help delineate the genetic, immune, and metabolic changes of this recently emerging clinical entity.

## Notes

Supplement. **eFigure 1.** Heel Involvement and Swelling of the Lesions **eFigure 2.** Ulceration **eFigure 3.** Histology of Disease Controls Including Perniosis and Leukocytoclastic Vasculitis **eFigure 4.** Capillaroscopy **eTable 1.** Dermoscopy Findings in Patients **eTable 2.** Laboratory Tests in Patients **eTable 3.** Histology Findings **eTable 4.** Capillaroscopy Findings
