# Supplementary material for: Bilateral Chilblain-like Lesions of the Toes Characterized by Microvascular Remodeling in Adolescents During the COVID-19 Pandemic
Source: JAMA Netw Open. 2021 Jun 10;4(6):e2111369. doi: 10.1001/jamanetworkopen.2021.11369 (PMC8193438; doi:10.1001/jamanetworkopen.2021.11369)
Supplement: Supplement. — eFigure 1. Heel Involvement and Swelling of the Lesions eFigure 2. Ulceration eFigure 3. Histology of Disease Controls Including Perniosis and Leukocytoclastic Vasculitis eFigure 4. Capillaroscopy eTable 1. Dermoscopy Findings in Patients eTable 2. Laboratory Tests in Patients eTable 3. Histology Findings eTable 4. Capillaroscopy Findings [file jamanetwopen-e2111369-s001.pdf]

Supplementary Online Content

Discepolo V, Catzola A, Pierri L, et al. Bilateral chilblain-like lesions of the toes characterized by microvascular remodeling in adolescents during the COVID-19 pandemic. *JAMA Netw Open*. 2021;4(6):e2111369. doi:10.1001/jamanetworkopen.2021.11369

eFigure 1. Heel Involvement and Swelling of the Lesions
eFigure 2. Ulceration
eFigure 3. Histology of Disease Controls Including Perniosis and Leukocytoclastic Vasculitis
eFigure 4. Capillaroscopy
eTable 1. Dermoscopy Findings in Patients
eTable 2. Laboratory Tests in Patients
eTable 3. Histology Findings
eTable 4. Capillaroscopy Findings

This supplementary material has been provided by the authors to give readers additional information about their work.

© 2021 Discepolo V et al. *JAMA Network Open*.

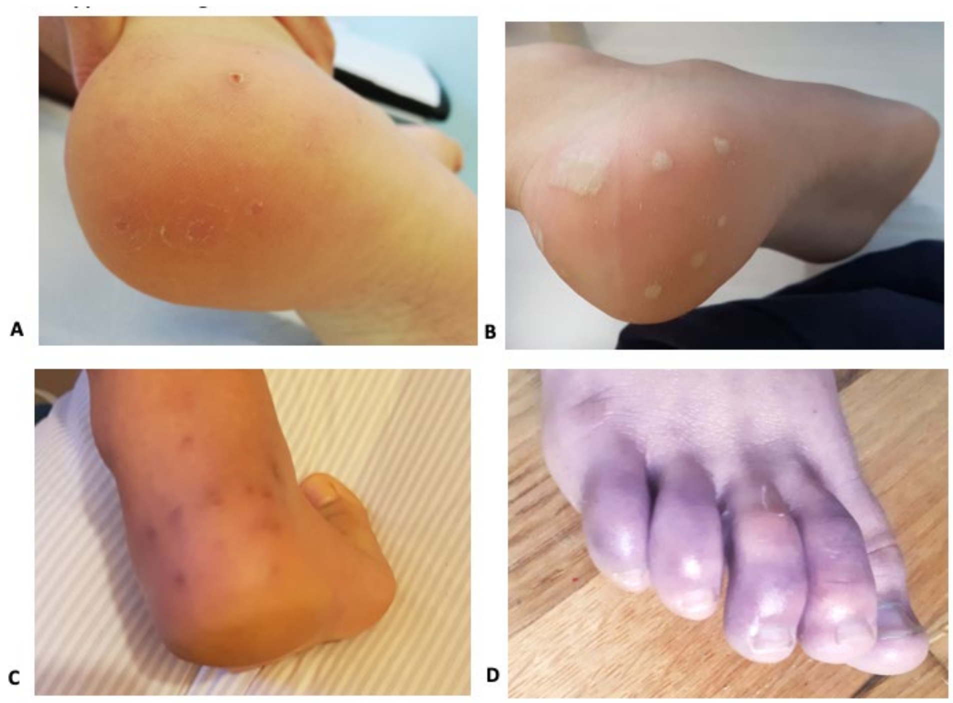

eFigure 1. Heel involvement and swelling of the toes. Lesions localized on the heels characterized few cases (A-C) The evolution of the lesions was characterized by desquamation (B). Swelling of the toes was also commonly reported in our cohort (D).

© 2021 Discepolo V et al. *JAMA Network Open*.

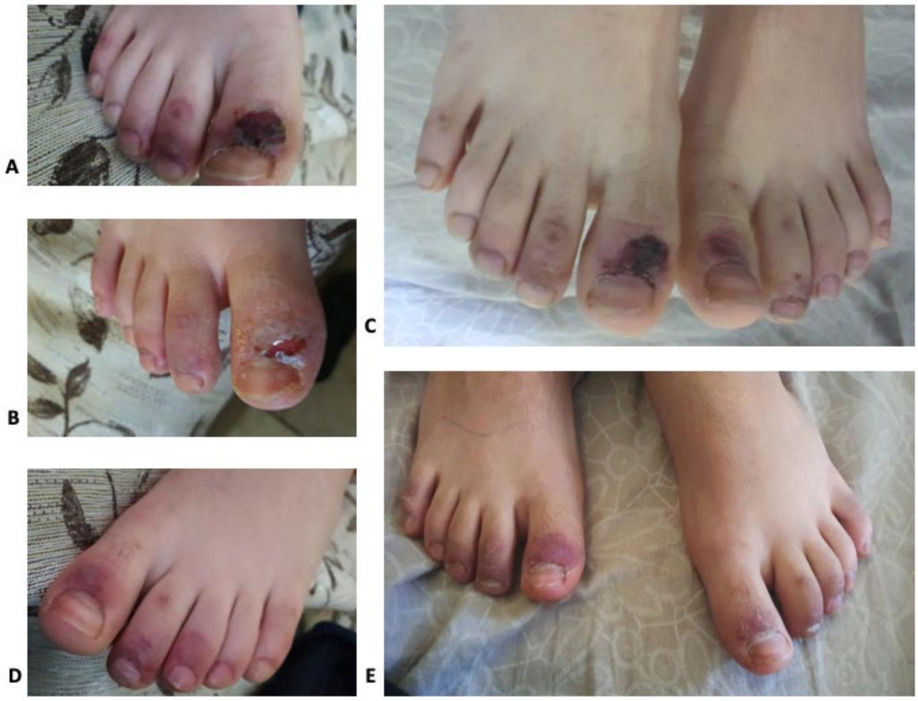

eFigure 2. Ulceration was a rare complication of the lesions. One representative case presented with ulceration of the right big toe. Evolution of the lesion over time is presented in the images (A-E).

© 2021 Discepolo V et al. *JAMA Network Open*.

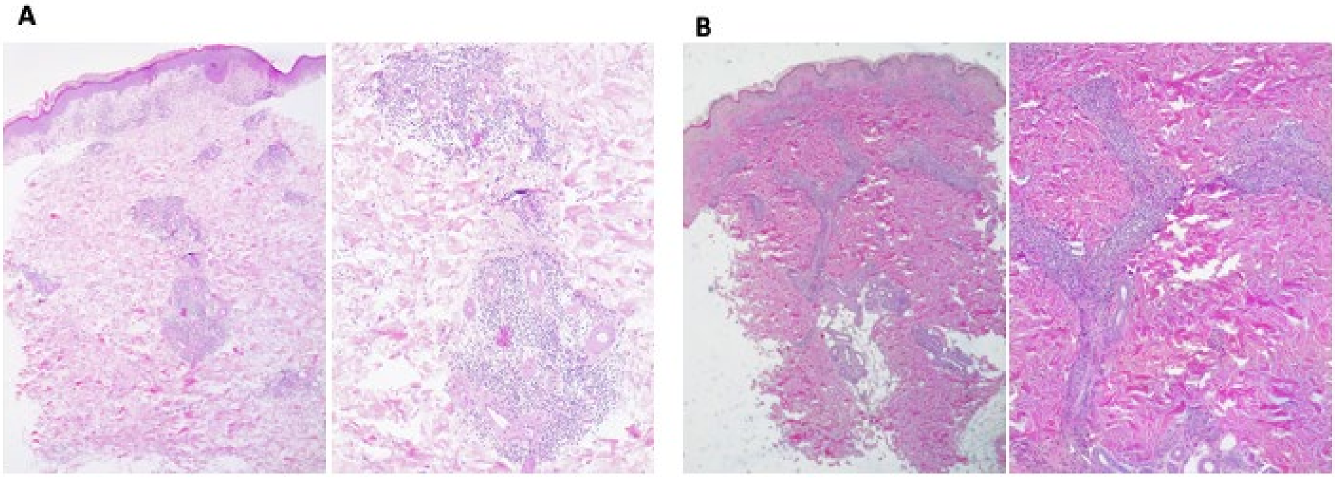

eFigure 3. Histology of disease controls including perniosis and leukocytoclastic vasculitis.

Haematoxilin and eosin staining of a representative case of perniosis (A) showing a moderate lymphocytic infiltrate with perivascular and peri-eccrine distribution and of leukocytoclastic vasculitis (B) characterized by an intense neutrophilic infiltrate involving the vessels wall. Original magnification 4X, left images and 10X, right.

© 2021 Discepolo V et al. *JAMA Network Open*.

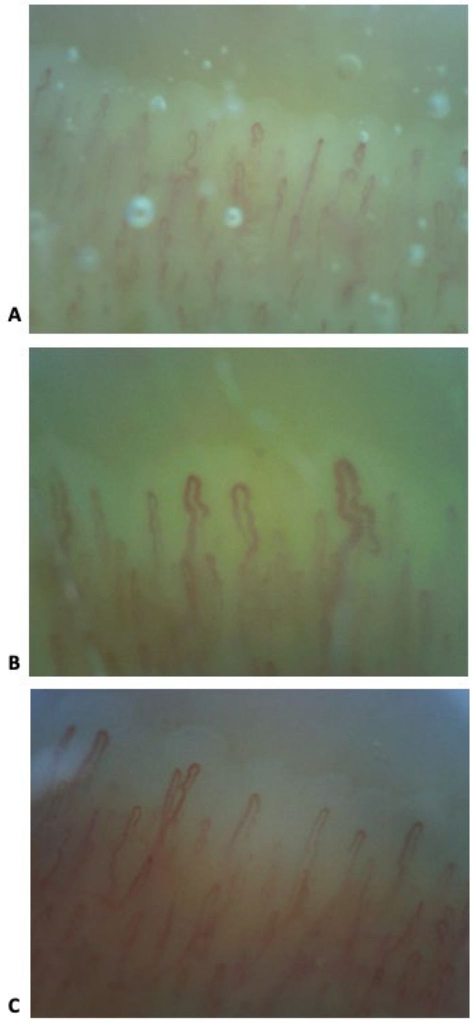

eFigure 4. Capillaroscopy reveals no systemic inflammatory process. Videocapillaroscopy images from three representative cases (A-C), showing normal microvascular architecture of the hands in most case or sparse ectasias.

© 2021 Discepolo V et al. *JAMA Network Open*.

eTable 1. Dermoscopy findings in patients.

		DERMOSCOPY T1						
**Patient Code**	**Onset to T1 (days)**	**Dermo T1**	**Red dots**	**White streak**	**Rosettes**	**Triad**	**Other pigmented areas**	**Desquamation**
1	13	Y	Y	Y	Y	Y	Y	Y
2	65	Y	Y	Y	Y	Y	N	N
3	24	Y	Y	Y	Y	Y	N	N
4	27	Y	Y	Y	Y	Y	Y	N
5	31	Y	Y	N	Y	N	Y	N
6	39	Y	Y	Y	N	N	Y	N
7	113	Y	Y	N	Y	N	N	N
8	34	Y	Y	Y	Y	Y	Y	N
9	83	Y	Y	Y	Y	Y	Y	N
10	52	Y	Y	Y	Y	Y	Y	N
11	46	Y	Y	N	Y	N	N	Y
12	45	Y	Y	N	N	N	N	N
13	57	Y	Y	Y	Y	Y	Y	N
14	54	N	NA	NA	NA	NA	NA	N
15	43	Y	Y	Y	Y	Y	Y	N
16	63	Y	Y	Y	Y	Y	N	N
17	12	Y	Y	N	N	N	N	N
	**DERMOSCOPY T2**							
	**T1 to T2 (days)**	**Dermo T2**	**Red dots**	**White streak**	**Rosettes**	**Triad**	**Other pigmented areas**	**Time to resolution (days)**
1	36	N	NA	NA	NA	NA	NA	49
2	22	N	NA	NA	NA	NA	NA	87
3	25	N	NA	NA	NA	NA	NA	49
4	25	N	NA	NA	NA	NA	NA	52
5	36	N	NA	NA	NA	NA	NA	67
6	36	N	NA	NA	NA	NA	NA	75
7	32	N	NA	NA	NA	NA	NA	145
8	35	N	NA	NA	NA	NA	NA	69
9	NA	N	NA	NA	NA	NA	NA	NA
10	37	N	NA	NA	NA	NA	NA	89
11	37	Y	N	N	N	N	N	83
12	35	Y	N	N	N	N	N	80
13	50	N	NA	NA	NA	NA	NA	107
14	NA	N	NA	NA	NA	NA	NA	NA
15	33	Y	N	N	N	N	1	76
16	28	Y	N	N	N	N	N	91
17	NA	N	N	N	N	N	N	NA

T1, enrollment visit. T2, follow-up visit. Y, yes. N, No. NA, not assessed.

© 2021 Discepolo V et al. *JAMA Network Open*.

eTable 2. Laboratory tests in patients.

	CBC						INFLAMMATION				
**PATIENT CODE**	**WBC Cell/µl**	**N**	**L**	**M**	**E**	**PLT Cell/µl**	**CRP mg/dl**	**PCT Ng/ml**	**ESR mm**	**Ferritin ng/ml**	**Ddimer mg/l**
1	5250	2860	1890	460	30	281	0.33	0.05	NA	28	0.09
2	4980	2320	2160	360	110	195	NA	0.05	20	36	0.25
3	5290	2760	1920	430	150	240	0.33	0.05	8	70	0.26
4	6800	3290	2690	460	290	266	0.33	0.05	10	47	0.22
5	8320	5050	2660	440	120	300	0.33	0.05	10	17	0.22
6	7010	3040	2740	430	730	286	0.33	0.05	18	61	0.13
7	4290	2390	1300	480	100	209	0.33	0.05	NA	27	0.16
8	4860	1720	2670	380	80	277	0.33	0.05	7	38	0.17
9	6240	2510	2680	310	470	221	0.33	0.05	NA	11	NA
10	5020	1750	2810	340	80	258	0.33	0.05	NA	46	0.14
11	5450	2610	2340	370	100	264	0.33	0.05	30	14	0.19
12	8690	3010	4520	520	70	247	0.33	0.05	15	42	0.26
13	3900	2120	1400	220	130	233	0.33	0.05	10	50	0.2
14	4420	NA	NA	NA	NA	222	0.44	0.03	NA	63.3	NA
15	4500	1690	2150	400	210	195	0.33	0.05	5	24	0.12
16	7380	5370	1570	380	30	215	0.7	0.05	22	28	0.38
17	7990	5090	2470	360	30	255	0.33	0.05	7	52	0.24

	IMMUNOLOGICAL LAB TESTS								
**PATIENT CODE**	**IgG g/l**	**IgA g/l**	**IgM g/l**	**ICC C1Q**	**ICC C3d**	**C3 g/l**	**C4 g/l**	**ANA**	**ENA**
1	10.1	1.09	0.97	0.67	2.49	**1.22**	0.21	NEG	NEG
2	10.6	2.24	0.69	1.3	2.73	**1.59**	0.25	NEG	NEG
3	9.82	1.07	0.82	0.72	1.81	**1.36**	0.21	NEG	NEG
4	12.6	2.33	0.84	1.81	2.41	**1.21**	0.19	NEG	NEG
5	10	0.78	0.74	6.3	1.2	**1.13**	0.17	NEG	NEG
6	9.92	3.25	1.27	2.79	2.48	**1.12**	0.18	NEG	NEG
7	8.55	1.45	1.28	0.41	1.48	**1.15**	0.19	NEG	NEG
8	11.7	1.8	0.5	NA	NA	**1.01**	0.21	NEG	NEG
9	11.7	2.3	2.09	NA	NA	**1.11**	0.15	NEG	NEG
10	12.4	1.04	3.28	1.08	1.28	**1.37**	0.15	NEG	NEG
11	9.45	1.25	0.83	5.03	1.28	**1.08**	0.11	NEG	NEG
12	9.57	1.75	0.38	9.53	1.98	**1.61**	0.31	NEG	NEG
13	10.2	0.99	0.42	0.27	0.6	**1**	0.15	NEG	NEG
14	6.76	2.05	0.55	NA	NA	**0.97**	0.2	NA	NA
15	10.7	1.5	0.92	0.63	2.72	**1.37**	0.15	NEG	NEG
16	8.79	0.95	1.74	2	2.1	**1.07**	0.15	NEG	NEG
17	14	3.26	1.96	3.21	2.44	**1.59**	0.3	NEG	NEG

WBC, white blood cells. N, neutrophils. L, lymphocytes. M, monocytes. E, eosinophils. PLT, platelets. CRP, C reactive protein. PCT, pro-calcitonin. ESR, Eritrocyte sedimentation rate. Ig, immunoglobulins. ICC, circulating immune complexes. C3, C4 complement fraction 3(normal value g/l 0.51-0.95), 4 (normal value 0.08-0.44). ANA, anti-nuclear antibodies. ENA, extractable nuclear antigens antibodies.

© 2021 Discepolo V et al. *JAMA Network Open.*

eTable 3. Histology findings.

Patient Code	Onset to T1 (days)	SKIN BIOPSY (Y/N)	Increased number vessels	Lobular organization	Endothelial hyperplasia	Lymphocytic perivascular infiltrate
1	13	N	NA	NA	NA	NA
2	65	Y	Y	Y	Y	Y
3	24	N	NA	NA	NA	NA
4	27	N	NA	NA	NA	NA
5	31	Y	Y	Y	Y	Y
6	39	Y	Y	Y	Y	Y
7	113	Y	Y	Y	Y	Y
8	34	Y	N	N	Y	Y
9	83	Y	Y	Y	Y	Y
10	52	N	NA	NA	NA	NA
11	46	N	NA	NA	NA	NA
12	45	Y	N	Y	Y	Y
13	57	Y	N	Y	Y	Y
14	54	Y	N	N	Y	Y
15	43	Y	Y	Y	Y	Y
16	63	Y	Y	N	N	Y
17	12	Y	N	Y	Y	N

T1, enrollment visit. T2, follow-up visit. Y, yes. N, No. NA, not assessed.

© 2021 Discepolo V et al. *JAMA Network Open*.

eTable 4. Capillaroscopy findings.

Patient Code	Vessels ectasia	Winding vessels
001	rare	N
002	N	N
003	N	N
004	rare	N
005	rare	N
006	frequent	Y
007	rare	N
008	rare	N
009	NA	NA
010	N	N
011	N	N
012	rare	Y
013	frequent	N
014	NA	NA
015	N	N
016	frequent	Y
017	NA	NA

Y, yes. N, No. NA, not assessed.

© 2021 Discepolo V et al. *JAMA Network Open*.
